# Online learning for crisis response: evaluating reach and perceived knowledge gains from the MOOC “Infection, Prevention, and Control of Acute Respiratory Infections for Healthcare Workers in Low- and Middle-Income Countries (IPC MOOC)”

**DOI:** 10.1186/s12909-025-07661-2

**Published:** 2025-08-07

**Authors:** Bernarda Espinoza-Castro, Verónica Encina, Marie Astrid Garrido, Fausto Ignatov Vinueza, Juan Pablo Piedra, Ximena Garzon-Villalba, Katja Radon

**Affiliations:** 1 CIH-LMU Center for International Health, LMU University Hospital Munich, Munich, 80336 Germany; 2 Institute and Clinic for Occupational, Social and Environmental Medicine, LMU University Hospital Munich, Munich, 80336 Germany; 3 Department of Health Sciences, University of Aysén, Eusebio Lillo, Coyhaique, 667 Chile; 4 Faculty of Health Sciences, Universidad de Las Américas (UDLA), Quito, 170513 Ecuador; 5 School of Public Health, Universidad San Francisco de Quito, Quito, 170901 Ecuador

**Keywords:** Infection prevention and control (IPC), Massive open online course (MOOC), Healthcare training, Crisis response training, Public health education

## Abstract

The COVID-19 pandemic had challenged healthcare systems worldwide, significantly affecting healthcare workers (HWs), particularly in low- and middle-income countries (LMICs). To address the urgent need for infection prevention and control (IPC) training among diverse healthcare roles, the Massive Open Online Course (MOOC) titled *"Infection, Prevention, and Control of Acute Respiratory Infections for Healthcare Workers in Low- and Middle-Income Countries (IPC MOOC)"* was developed and implemented in Ecuador. This study aimed to evaluate reach and perceived knowledge gains from the IPC MOOC, focusing on whether successful course completion was influenced by sociodemographic factors or occupational roles (manual vs. intellectual work) and whether satisfaction and perceived learning outcomes differed between these groups. The IPC MOOC was developed through an interdisciplinary collaboration involving experts from the Center for International Health at the LMU Munich University Hospital (CIH^LMU^) and their partners in Latin America. It utilized problem-based learning and interactive scenarios to teach IPC principles in the context of COVID-19. The course was offered to all Ecuadorian healthcare workers between August and December 2021, with a total of 3498 participants enrolling. Data were collected through registration and post-MOOC surveys and analysed using Chi-squared and Mann-Whitney U tests to assess the influence of sociodemographic and occupational factors on course completion and satisfaction, and to compare perceived learning outcomes between manual and intellectual workers. Of the enrolled participants, 75% completed the IPC MOOC successfully, with no significant differences in completion rates based on gender, region, or occupation. Among the 809 participants who completed the post-course survey, 80% reported high satisfaction (on a scale from 0 to 100%) with the course, and 95% would recommend it to colleagues. There was a small but statistically significant difference in perceived knowledge before the course between manual and intellectual workers (3.41 vs. 3.57 on a 5-point Likert scale; *p*=0.02), but post-course perceived knowledge was similar for both groups (4.08 vs. 4.14 on the same Likert scale; *p*=0.41). The IPC MOOC demonstrated to be an effective and accessible training tool, bridging knowledge gaps across diverse healthcare roles and promoting equitable access to IPC education. The high completion and satisfaction rates indicate its potential as a scalable educational intervention in health crisis.

**Supplementary Information:**

The online version contains supplementary material available at 10.1186/s12909-025-07661-2.

## Introduction

The COVID-19 pandemic had significantly impacted healthcare systems worldwide, placing immense strain on the health workforce, one of the six essential building blocks of health systems [1]. The scale of the outbreak and the nature of the public health response have varied from country to country, with contextual factors influencing the extent to which healthcare workers (HWs) were exposed to pandemic-related stressors [2]. In the Americas, between the start of the pandemic and the end of 2021, there were at least 2.4 million reported cases of COVID-19 among HWs, resulting in about 13,000 deaths. These cases accounted for 16% of the estimated 15 million HWs in the region [3, 4].

This drastic increase in infections forced changes in the management of healthcare facilities. Clinical staff were assigned as frontline personnel, while non-clinical staff, such as administrative staff, maintenance workers, food service workers, custodians, and support personnel, took over the tasks of those deployed to the front lines [5, 6]. Additionally, some HWs had to work with COVID-19 patients without having a professional background or training in infection prevention and control (IPC), and qualified professionals did not have the time to properly train their colleagues [7, 8]. The varying nature of these roles, combined with delayed or insufficient IPC guidelines, created significant challenges and uncertainty for HWs [7].

These challenges underscore the critical role of infection prevention and control (IPC) measures in protecting both healthcare workers and patients. However, the lack of adequate IPC training and resources exacerbated the risks faced by HWs during the pandemic. Recognizing these challenges, the World Health Organization (WHO) highlights the importance of IPC, defining it as “a clinical and public health specialty that uses a practical, evidence-based approach to prevent patients, health workers, and visitors to healthcare facilities from being harmed by avoidable infections, acquired during the provision of healthcare services” [9]. Hence, it has been suggested that providing proper IPC training and adequate personal protective equipment (PPE) to all HWs is indispensable for ensuring a safe workplace and maintaining workforce availability in the long run [10]. Several studies conducted during the COVID-19 pandemic had reported various issues related to IPC and PPE training for healthcare workers. These issues include a lack of or insufficient training, training that is not easily accessible or adaptable to the demanding schedules of healthcare workers, and training that excludes cleaners, porters, kitchen staff, and other support personnel [10–16]. For instance, a qualitative study by Jeleff et al. reported that cleaning staff experienced fear of being infected by SARS-CoV-2, primarily due to a lack of information regarding transmission pathways [7].

Given the rapid spread of the virus and the urgent need for up-to-date knowledge and practices, several organizations switched from in-person IPC training to online learning. This shift required an expansion of online learning opportunities for HWs such as the massive open online courses (MOOCs) [17]. MOOCs are advantageous because they can reach large and diverse groups from various geographic and socioeconomic backgrounds [18]. They are often offered for free or are relatively inexpensive compared to traditional forms of education and can enhance learner engagement and motivation if they include interactive elements [19], making them suitable for emergency response situations [18–20]. However, MOOCs are also criticized for having low completion rates [21], being difficult for learners with limited technological proficiency or poor internet connectivity, and offering limited direct interaction with instructors [22].

Despite these challenges, MOOCs remain a valuable tool for rapidly disseminating critical knowledge, particularly in crisis situations. Building on this potential, the MOOC: Infection, Prevention, and Control of Acute Respiratory Infections for Healthcare Workers in Low- and Middle-Income Countries (IPC MOOC) was developed to equip healthcare workers worldwide with essential IPC knowledge to mitigate the spread of SARS-CoV-2 in healthcare settings. In this context, the IPC MOOC was implemented in Ecuador, targeting HWs in public healthcare facilities. Ecuador was selected as a case study due to its persistently high rates of COVID-19 infections among HWs [23], as well as the significant challenges in providing timely and accessible training, particularly in rural areas [24]. Another key factor was the existing disparities in healthcare training across socioeconomic groups, driven by differences in education and income levels [23, 25, 26]. Additionally, the widespread availability of Internet access in many healthcare facilities provides an opportunity to mitigate these gaps through online educational platforms, enhancing equitable access to medical training [23, 27].

To evaluate the potential and success of the IPC MOOC, this study aimed to answer two key research questions: 1) Did the successful completion of the IPC MOOC depend on sociodemographic factors or occupation (manual work/intellectual work)? and 2) Were satisfaction levels and perceived learning outcomes equally well suited for the target group?

## Methods

### MOOC development and delivery

The MOOC: Infection, Prevention, and Control of Acute Respiratory Infections for Healthcare Workers in Low- and Middle-Income Countries (IPC MOOC) was developed through an iterative process involving regular Zoom meetings and email exchanges between the course lead and the project team. The team was characterized by its interdisciplinary nature (nursing (VE, MAG), sociology (FIV), medicine (BEC), and occupational epidemiology (KR)) and its international composition (Germany, Ecuador, and Chile), with experience in blended learning education from the Center for International Health at the University Hospital of LMU Munich (CIH^LMU^) within the OH-TARGET project (One Health Training And Research Global NETwork), founded by the German Federal Ministry for Economic Cooperation and Development (BMZ) and the German Academic Exchange Service (DAAD).

The IPC MOOC is offered via the Learning Management System “CIH^LMU^ Moodle” (https://cih-moodle.med.lmu.de/course/view.php?id=246). It was provided in Spanish and has an estimated workload of approximately 20 hours. The MOOC was developed using a problem-based learning methodology. It consists of nine mandatory units and one optional unit. Each unit contains animated and interactive scenarios introducing specific risk situations that healthcare workers might have faced daily during the COVID-19 pandemic as an example of an acute respiratory infection. These scenarios follow the story of Mr. Fountain, a patient who begins his journey at a primary healthcare facility after showing symptoms of an acute respiratory infection. As his condition worsens, he is transferred to a hospital, where he progresses through the emergency room, hospitalization, and Intensive Care Unit (ICU), highlighting the importance of proper IPC measures and mental health strategies for healthcare workers (Table 1) in each setting, taking into consideration their particularities from the HW’s perspective.

**Table 1:** Didactic concept of the IPC MOOC

Units	Scenarios	Contents	Learning objectives (at the end of this chapter, participants will be able…)
**1. COVID-19 in a Nutshell**	This scenario describes the nature of SARS-CoV-2 and COVID-19	This unit includes information about the nature of SARS-CoV-2 and COVID-19, including its pathophysiological mechanisms, transmission, symptoms, diagnosis, treatment, and prevention	Identify the general pathophysiological mechanisms, modes of transmission, and symptoms of acute respiratory infections (ARIs), with a specific focus on COVID-19
**2. Occupational hazards and risks & IPC**	This scene takes place in a primary healthcare facility in a small suburban community, where a patient, Mr. Fountain, arrives after experiencing symptoms of an acute respiratory infection	The main contents cover concepts of risk and hazard, types of risk, risk management through the Hierarchy of Controls, and Infection Prevention and Control (IPC) measures, including engineering and administrative controls, standard precautions, hand hygiene, and respiratory hygiene	- To distinguish between risk and hazard in the workplace and categorize Infection Prevention and Control (IPC) measures according to the risk management hierarchy of controls - To identify the steps, timing, and elements used during hand hygiene, differentiate when handwashing and alcohol-based handrub is appropriate, and recognize the “5 moments for Hand Hygiene” promoted by WHO
**3. IPC in patient transportation**	Mr. Fountain’s medical conditions deteriorate at the primary health and must be transported to a hospital. This scenario plays out in the ambulance, where participants learn to recognize the standard precautions during and after patient transport	The main contents focus on control measures in patient transport, precautions to prevent pathogen transmission by contact and droplets, and post-transport cleaning and disinfection	To identify and implement standard and additional measures to prevent the transmission of pathogens and control the risk of infection during and after patient transport and recognize the importance of post-transport cleaning and disinfection practices
**4. Correct use of Personal Protective Equipment (PPE)**	In this scenario, Mr. Fountain arrives at the hospital and is received in the emergency room. This scenario focuses on the correct use of PPE	This unit is devoted to the correct use of personal protective equipment (PPE) in the context of COVID-19 (types of PPE, and PPE donning and doffing) as control measures	To distinguish the required PPE according to the risk in the job or activity in the context of COVID-19 and to demonstrate the PPE donning and doffing process
**5. IPC in laboratory and imaging**	Mr. Fountain has been hospitalized due to difficulty breathing. As part of diagnosing an acute respiratory infection, laboratory tests and X-rays are conducted. This scenario highlights the IPC measures in laboratory and imaging services	The main content includes preventive measures for collecting and packing/shipping COVID-19 samples, as well as the classification of laboratories according to biosafety levels. It also covers preventive measures for radiology and imaging service workers.	To list the infectious agent category, describe the packaging type and labelling for shipping samples, identify laboratory types according to biosafety levels, and recognize the administrative measures and required PPE for radiology and imaging service workers.
**6. Terminal cleaning of rooms and laundry in the context of COVID-19**	Mr. Fountain has been hospitalized in an area reserved for patients with acute respiratory infections. After a few days, his condition worsens, and he is transferred to the Intensive Care Unit (ICU). The room where Mr. Fountain was staying now needs to be prepared for other patients. This scenario highlights the importance of proper cleaning and laundry services in hospital rooms	The main contents of PCI - Standard preventive measures in the context of COVID-19 include guidelines for laundry and terminal cleaning in hospitalization services	To describe prevention measures for hospital laundry service workers and identify prevention measures for cleaning and sanitation workers, including the steps of terminal cleaning in a patient room diagnosed with COVID-19
**7. IPC in the Intensive Care Unit (ICU)**	Mr. Fountain has spent his first night in the ICU after his condition worsened. This scenario focuses on the daily activities and challenges faced by healthcare workers in an ICU setting	The main contents include preventive measures according to PCI in an ICU in the context of COVID-19, with an emphasis on precautions during high-risk aerosol-generating procedures	To identify prevention and control measures in the ICU in the context of COVID-19
**8. Handling Dead Bodies in the context of COVID-19**	During the night, a patient has passed away in the ICU. Dr. Adams has been called to transfer the body to the mortuary department. This scenario outlines the IPC measures necessary when handling and disposing of the body of a deceased COVID-19 patient	The main content includes management of contaminated/suspected COVID-19 corpses according to infection control protocols	To list the preventive measures for mortuary’s workers (staff manipulating bodies/dead by COVID-19) and to review the right PPE donning and doffing in thanatology
**9. Recommendations for Mental Health Care in the context of COVID-19**	To support the staff, the hospital implemented various strategies to address mental health concerns and prevent psychosocial risks, overseen by a psychologist from the Human Resources Department. This scenario describes self-care behaviours that promote good mental health	The main contents include recognizing symptoms related to psychosocial risk and promoting self-care behaviours to maintain good mental health among health workers during the pandemic.	To recognize major mental health risks and hazards among healthcare workers in emergency situations caused by COVID-19 and to identify healthy practices to prevent mental health problems in such scenarios
**10. Additional Content: How to teach a practical skill effectively?**	Mr. Fountain has responded well to treatment and was discharged after a hospital stay of a couple of weeks. As a local school teacher, he reflects on how he can teach his students proper hand hygiene to help prevent acute respiratory infections	The main content includes "The Four-Step Method of Teaching a Skill by Rodney Peyton"	To design a teaching plan for hand hygiene (washing or disinfection) or PPE donning and doffing to students or colleagues using Rodney Peyton's clinical skills teaching methodology

Participant progress was tracked on the CIH^LMU^ Moodle platform, allowing for the verification of course activity completion and calculation of final grades. The Moodle-based format allowed participants to access the course from both computers and portable devices alike. This ensured greater accessibility and flexibility, accommodating different user preferences and device availability. Participants who met the course requirements received a certificate of participation from CIH^LMU^. The requirements to complete the course and receive a certificate were: 1) successful completion of the nine units with a minimum score of 60% on the tasks, and 2) passing the Final Assessment with a minimum score of 60%

### Study participants and setting

Between August and December 2021, the Ecuadorian Ministry of Health, in collaboration with CIH^LMU^, offered the IPC MOOC to all healthcare workers in the public health sector in Ecuador, as part of a support initiative during the COVID-19 pandemic. The course was recommended to doctors, nurses, medical assistants, administrative staff, porters, caterers, and maintenance and cleaning workers. A total of 3,498 participants registered for the course and completed the registration survey, out of an estimated 90,000 healthcare workers in Ecuador, representing approximately 4% of the country’s public health workforce [28]. Of those registered, 2677 participants successfully completed the course by achieving a minimum score of 60% on all evaluations and assignments. Upon course completion, a post-MOOC survey was offered to assess participants'perceptions of their knowledge before and after the course, as well as their feedback and overall satisfaction with the course. The voluntary post-MOOC survey was completed by 809 participants who had successfully finished the course.

### Data collection

#### Registration survey

The data for this study were collected at two key points: during initial registration and after completion of the MOOC. All surveys were administered in Spanish to ensure accessibility for the target audience. In both surveys, all participants provided informed consent to participate in the study prior to data collection. The registration survey included sociodemographic information such as gender (male, female, other), health institutions (The Ministry of Public Health Ecuador, the Ecuadorian Institute of Social Security (IESS), the Institute of Social Security for the Armed Forces (ISSFA), and Social Security Institute of the National Police (ISSPOL)), and region of work (Amazon region, Coastal and Galápagos region, and Andean region). Current occupation was also assessed and classified according to the International Standard Classification of Occupations (ISCO-08), divided into two categories: manual workers and intellectual workers [29]. “Manual workers” included individuals whose jobs required either no training, specific job-related training, or a secondary school certificate. “Intellectual workers” refers to participants whose positions require university education.

#### Post-MOOC survey

The post-MOOC survey consisted of 26 questions assessing the first level of Kirkpatrick’s evaluation model [30]. The first section of this survey assessed participants'perception of their prior knowledge before starting the course on a scale from 0 (very low knowledge) to 5 (high knowledge). The feedback section included eight questions evaluating satisfaction with the course, such as content and structure, the time required, the use of interactive videos, the final assessment, the automatic registration process, the support provided by the tutors, and overall satisfaction. Answers were assessed on a 5-point Likert scale ranging from “not at all satisfied” to “highly satisfied” (0 to 5). Finally, the survey measured participants'perception of their level of knowledge after completing the course, again using a scale from 0 (very low knowledge) to 5 (high knowledge).

### Data analysis

Data analysis was performed using SPSS® version 25.0 (IBM, Armonk, NY, USA) to address the following research questions:

1. Did the successful completion of the IPC MOOC depend on sociodemographic factors or occupation (Manual work/intellectual work)?
2. Did the level of satisfaction and perceived learning vary between HWs who perform manual work and those who perform intellectual work?

For the first question, sociodemographic characteristics and occupation between participants who passed the course and those who did not pass the course were compared using Chi-squared test. The nominal and ordinal variables were presented as absolute and relative frequencies. For the second question, a bivariate analysis using the Mann-Whitney U test was performed to assess the difference in satisfaction and perceived learning between professionals who perform manual work and those who perform intellectual work.

## Results

All 3498 enrolled participants were Ecuadorians, from the Coastal and Insular regions (46%) and the Andean region (44%). More than half of the participants were female (58%). About 57% of the participants were intellectual workers (doctors (31%) and nurses (20%) representing the largest groups). With respect to the type of health institution, health-workers employed by the Ministry of Public Health were the largest group (40%).

A total of 75% of participants completed the course requirements and passed the course. There was no statistically significant difference in sociodemographic between participants who passed the course and those who did not (Table 2).

**Table 2:** Descriptive analysis of sociodemographic characteristics and course completion status of 3498 IPC MOOC participants

Variables		Course completion				pχ^2^
		No^a^		Yes		
		n	(%)	n	(%)	
**Gender**	Female	503	(58.2)	1532	(58.4)	0.90
	Male	362	(41.8)	1092	(41.6)	
**Place of work**	Amazonian region	83	(9.7)	226	(8.7)	0.56
	Cost & Galapagos region	393	(45.6)	1206	(46.4)	
	Andean region	384	(44.7)	1172	(45.0)	
**Occupation**	Intellectual workers^c^	335	(56.4)	780	(56.3)	0.45
	Manual workers^b^	258	(43.7)	768	(43.1)	
	ISSFA^d^	246	(31.5)	705	(29.4)	0.57
**Health Institutions**	IESS^e^	159	(20.4)	541	(22.5)	
	Ministry of Public Health	318	(40.7)	978	(40.7)	
	ISSPOL^f^	58	(7.4)	178	(7.4)	

^a^Participants who either dropped out of the MOOC or did not achieve the passing grade

^b^Manual workers: participants whose jobs required either no training, specific job-related training, or a secondary school certificate

^c^Intellectual workers: participants whose positions require higher education (university)

^d^Institute of Social Security for the Armed Forces

^e^Ecuadorian Institute of Social Security

^f^Social Security Institute of the National Police

Of the 809 participants who completed the post-IPC MOOC questionnaire, approximately 63% were intellectual workers. Half of the participants completed the course using a computer only, while 15% used both computers and smartphones. Most of the respondents (80%) reported high satisfaction with the course content, structure, and interactive videos, while 95% stated they would recommend the course to their colleagues. The overall quality of the course was rated with a median score of 4.31 on a scale from 0 (poor) to 5 (excellent) (25th percentile 3.92; 75th percentile 4.67).

**Fig. 1:**
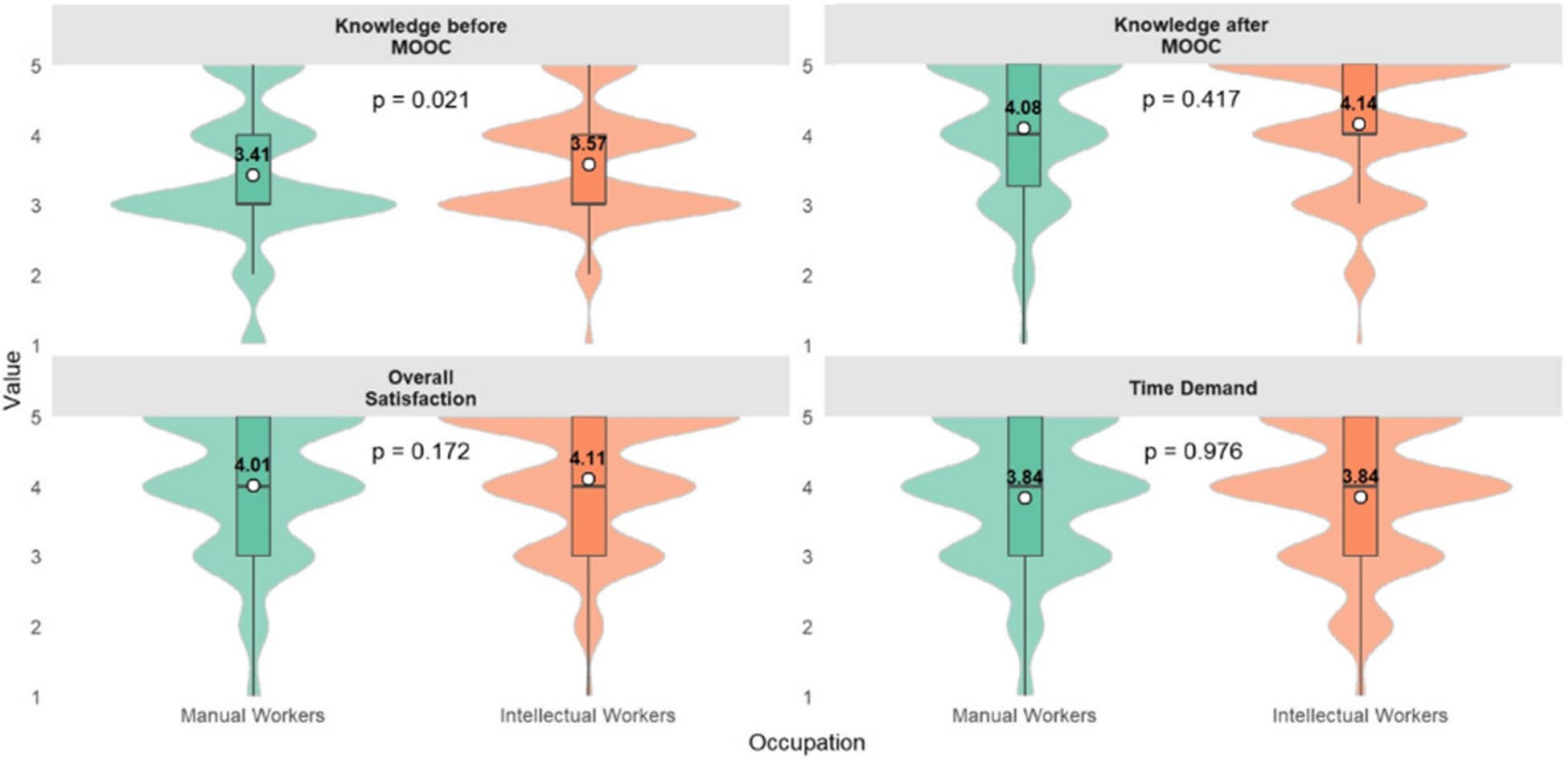
Evaluation of IPC MOOC by occupation (Manual: *n*=297 vs. Intellectual: *n*=512)- knowledge perception, overall satisfaction, and time demand

There was a small but statistically significant difference in the perception of the level of knowledge before the IPC MOOC between manual workers and intellectual workers (3.41 vs. 3.57; *p*=0.02). By the end of this course, the perceived knowledge increased up to 4.08 in the group of manual workers and to 4.14 in the group of intellectual workers (*p*= 0.41). No significant differences were observed between the two groups in overall satisfaction or perceived time demand (Fig. 1).

## Discussion

To the best of our knowledge, this is the first study to evaluate an IPC-focused MOOC designed for healthcare workers across all occupational roles in low-resource settings during the COVID-19 pandemic. Our results indicate that the intervention achieved its intended objectives.

The urgency of IPC training became evident as studies highlighted how task shifting, driven by shortages of healthcare workers, particularly in low- and middle-income countries, created a pressing need to equip all roles of health personnel with adequate IPC skills [31–33]. In many cases, non-clinical staff were reassigned to medical roles without adequate preparation [5, 6], underscoring the need for accessible IPC education [7, 8]. Findings from Tomczyk et al. reinforce this necessity, demonstrating that IPC training benefits all staff categories, including those with prior knowledge [34]. Furthermore, they emphasize that in complex emergencies, IPC training must extend beyond traditional frontline workers, as the definition of those at risk broadens [34]. Our IPC MOOC effectively addressed these challenges by providing training that was independent of socio-demographic factors or occupational roles. A statistically significant difference in perceived knowledge was observed between manual and intellectual workers before the course. However, both groups reported similar knowledge levels after completion, suggesting that the MOOC helped reduce knowledge disparities and equipped all healthcare workers with essential IPC competencies.

Another major factor contributing to the course’s effectiveness was the high global demand for IPC knowledge during the pandemic and the advantages of the MOOC format [18, 35, 36]. Several studies have highlighted inadequate IPC training, particularly among support staff such as cleaners, porters, and administrative personnel [7, 11, 13, 15, 16]. For example, Elhadi et al. (2020) found that 53% of healthcare workers in Libya lacked sufficient IPC training, partly due to the limitations of in-person training [37]. MOOCs addressed this gap by providing a scalable and accessible learning platform, reaching a broad audience regardless of location [18, 35] and taking into consideration accessibility aspects regarding device preferences and availability. Their flexible format allowed for rapid knowledge dissemination, ensuring that healthcare workers could receive essential IPC training even when traditional in-person methods were not feasible [35, 36]. This MOOC serves as an example of rapid reach, having engaged 3,498 healthcare workers within just five months.

In addition to the course format, a further strength in the Ecuadorian context is the relatively high level of internet connectivity compared to many other low- and middle-income countries. According to the Pan American Health Organization (PAHO), 76.2% of Ecuador’s population had an internet connection in 2021 [27]. While limited internet access is often cited as a barrier to MOOC participation in many regions, in Ecuador it functioned as an enabling factor, facilitating broader engagement with the IPC MOOC across diverse healthcare worker groups.

This broad access to connectivity, combined with the overall effectiveness of the IPC MOOC, might contributed to the high levels of engagement and completion observed in the course. Approximately, 80% of participants expressed satisfaction with the course content, structure, and interactive videos. This satisfaction likely contributed to the course’s 75% completion rate, which exceeded the typical MOOC completion rates of 5%–10% [18, 21] and was even higher than OpenWHO courses (45.9%), Murugesan et al. (53%), and Goldin’s courses (56%) [35, 38, 39]. The urgency of acquiring crisis-related knowledge and support from the Ecuadorian Ministry of Health likely played a role in this high engagement. Additionally, Maxwell’s findings suggest that the inclusion of animated videos, such are those implemented in the IPC MOOC, might improve interactivity and reduce learning time [18, 19].

Despite these strengths, this study has some limitations. First, a potential completion bias may have been introduced, as the evaluation included only participants who successfully completed the course. Additionally, the relatively low response rate to the post-MOOC survey may have introduced response bias, limited the representativeness of participants’ feedback.

Second, due to the emergency conditions under which the IPC MOOC was implemented, a pre-course assessment was not conducted to minimize the burden on healthcare workers. Instead, participants retrospectively rated their prior knowledge in the post-course survey, relying on self-perception rather than objective measures. While this approach enabled timely data collection during a crisis [40], it may have introduced recall or response-shift bias, limiting the reliability of conclusions about actual knowledge acquisition. Future evaluations should incorporate a pre-post design to more accurately measure learning outcomes.

Third, the study focused exclusively on short-term outcomes based on self-reported data collected immediately after course completion. As a result, mid- and long-term effects such as sustained knowledge retention and the application of IPC practices in daily professional settings were not assessed. Future research should include longitudinal follow-ups with larger and more diverse samples to evaluate the persistence and real-world impact of the training.

Fourth, because the IPC MOOC was implemented within the specific healthcare and sociocultural context of Ecuador, the generalizability of the findings to other low- and middle-income countries (LMICs) may be limited. Differences in healthcare infrastructure, digital access, and pandemic response strategies could influence both the course’s accessibility and its effectiveness in other contexts.

Finally, although tutors were available to answer questions through online platforms, the absence of face-to-face interaction may have reduced participant engagement and limited opportunities for direct clarification and peer exchange [41].

## Conclusion

This study highlights the potential of MOOCs as an effective training tool for healthcare workers across various roles, particularly in crisis situations. The IPC MOOC served as an innovative educational response to the COVID-19 pandemic, ensuring rapid and scalable dissemination of essential IPC knowledge. It indicates a strong contribution to perceived knowledge enhancement among healthcare workers in a timely manner, 3498 participants in five months.

A key strength of the course was its accessibility in low-resource settings, where healthcare workers often face barriers to training opportunities, such as availability of time, resources and lack of strategic training policy. The IPC MOOC remains open to international health workers for infectious disease prevention, with free access available in English, Portuguese, and Spanish (https://www.cih.lmu.de/education/short-term-courses/infection-prevention-and-control-ipc-of-acute-respiratory-infections-aris-for-healthcare-workers). The ability of MOOCs to deliver cost-effective training and facilitate widespread knowledge dissemination was a key factor in the Ecuadorian Ministry of Health’s endorsement of the initiative.

## Supplementary information

Supplementary Material 1.

## Data Availability

No datasets were generated or analysed during the current study.

## Abbreviations

HWs: Healthcare workers
ICU: Intensive Care Unit
IESS: Ecuadorian Institute of Social Security (Instituto Ecuatoriano de Seguridad Social)
IPC: Infection prevention and control
ISCO-08: International Standard Classification of Occupations
ISSFA: Institute of Social Security for the Armed Forces (Instituto de Seguridad Social de las Fuerzas Armadas)
ISSPOL: Social Security Institute of the National Police (Instituto de Seguridad Social de la Policía)
LMICs: Low- and middle-income countries (LMICs).
MOOC: Massive Open Online Course
PPE: Personal protective equipment
WHO: World Health Organization
