# Supplementary figures and images for: Online learning for crisis response: evaluating reach and perceived knowledge gains from the MOOC “Infection, Prevention, and Control of Acute Respiratory Infections for Healthcare Workers in Low- and Middle-Income Countries (IPC MOOC)”
Source: BMC Med Educ. 2025 Aug 7;25:1150. doi: 10.1186/s12909-025-07661-2 (PMC12330176; doi:10.1186/s12909-025-07661-2)

**Annex**

**Figure 2: Feedback evaluation of the IPC MOOC**


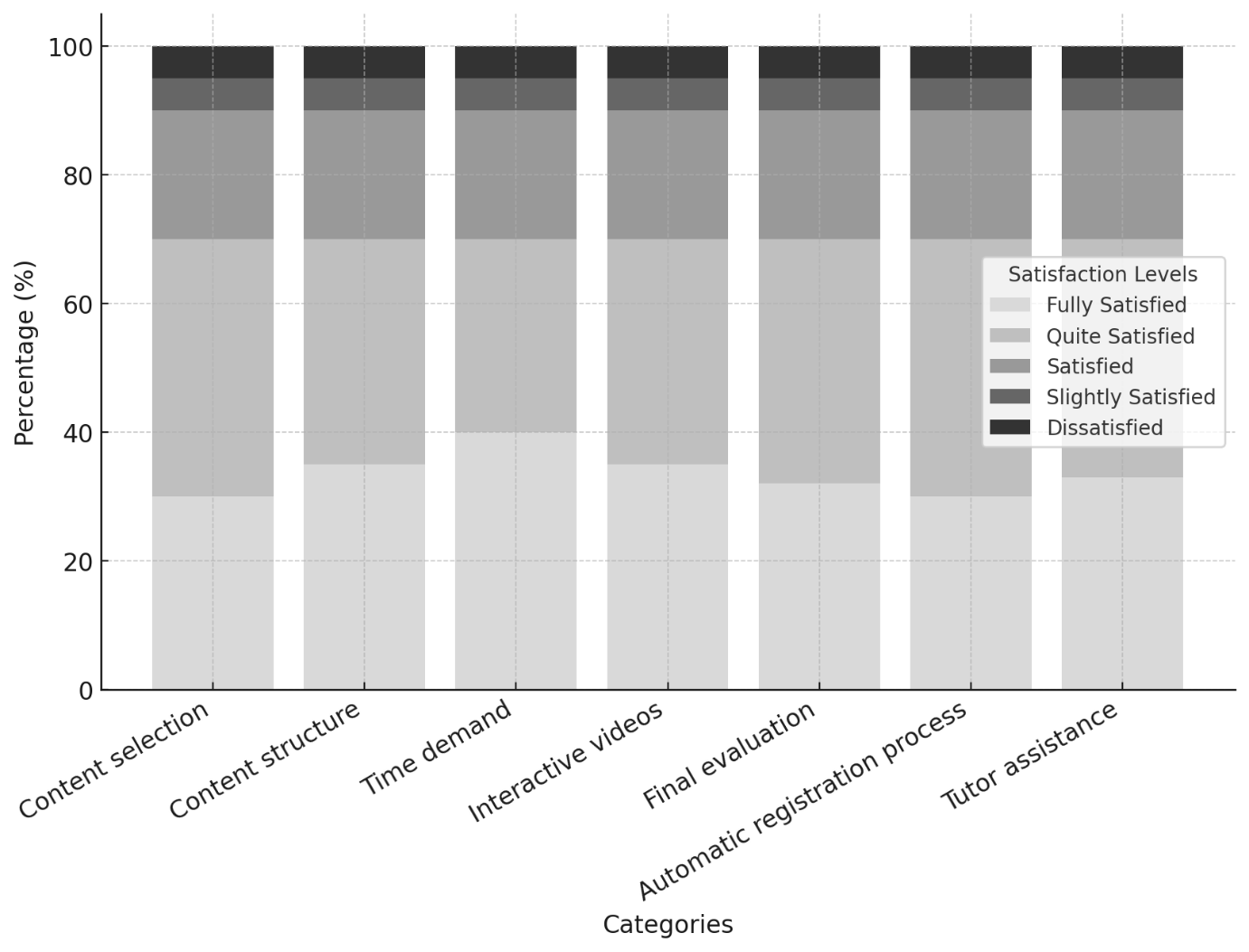

Supplement: Supplementary file 1 — Supplementary Material 1. [file 12909_2025_7661_MOESM1_ESM.docx]
